# Potential, Challenges, and Threats for the Application of New Breeding Techniques by the Private Plant Breeding Sector in the EU

**DOI:** 10.3389/fpls.2020.582011

**Published:** 2020-09-25

**Authors:** Petra Jorasch

**Affiliations:** Manager Plant Breeding Innovation Advocacy, Euroseeds, Brussels, Belgium

**Keywords:** plant breeding, genome editing, Crispr, GMO, regulation, mutagenesis, biotechnology, seed industry

## Abstract

Reconciling sustainability with agricultural productivity in the face of climate change relies strongly on the development of resilient, high-yielding crops of superior nutritional value that can be grown more resource efficiently. Therefore, innovation in plant breeding has gained unprecedented importance. Plant breeding depends upon genetic variability within crops and their relatives as a basis for developing new plant varieties with improved characteristics. Plant breeders are continuously integrating the latest methods in plant biology and genetics into their breeding toolbox to more efficiently use existing diversity but also to induce new genetic variation. Over the past years, ever more precise and efficient plant breeding methods have been developed. This plant breeding innovation leap is based on an in-depth understanding of plant genomes and refinement of breeding methods, enabling more efficient, more precise and faster progress in achieving the desired breeding goals. Consequently, these plant breeding innovations are rapidly being developed and utilized internationally and across the seed sector, public and private research, plant species and markets. The results of a survey among 62 private plant breeding companies conducted by Euroseeds and presented in this publication confirm the enormous interest of companies in using new breeding techniques (NBTs) for a wide range of crop species and traits and the negative impact of the current regulatory situation in the EU on companies’ decisions for investments in NBT-related R&D activities for the EU market and beyond.

## Introduction

Europe’s seed sector, technology developers and public researchers are global leaders in developing improved plant breeding methods (Lusser et al., 2011). The sector is highly innovative and invests up to 20% of its turnover in research and development, to constantly provide farmers with the best varieties that fit the needs of a highly productive and sustainable agriculture and respond to consumer demands. With an increased understanding of plant biology and plant genes, plant breeders have constantly improved their breeding tools to include a wide variety of breeding methods. The development of more recent plant breeding methods has not led to a complete replacement of the older ones. Depending on the challenges plant breeders must tackle, they must be able to choose the tools that enable them to reach their breeding goals in the most efficient and specific way. NBTs (as defined in Lusser et al., 2011 including genome editing and targeted mutagenesis) have raised high interest worldwide among scientists and breeders as new tools to increase breeding efficiency especially with emergence of the CRISPR technology in 2012 (Zhang et al., 2020).

However, the regulatory burden on NBTs is high in Europe. Regarding mutagenesis breeding the ruling of the European Court of Justice (2018) confirmed that:

- organisms obtained by all means of mutagenesis must be considered to be Genetically Modified Organisms (GMOs) as defined in article 2(2) of Directive 2001/18/EC (GMO Directive),
- the mutagenesis exemption only applies to methods of mutagenesis which have conventionally been used in a number of applications and have a long safety record. Organisms obtained by applying exempted methods are considered GMOs exempted from GMO regulation. NBTs are not considered exempted methods of mutagenesis.

Consequently, the prohibitive compliance requirements of the GMO regulations relative to the value of commodity crops effectively cut Europe’s breeders off from scientific progress and puts them as well as farmers, processors, traders, and consumers at a competitive disadvantage to regions with more enabling regulations. In addition, it creates legal uncertainty for market operators. Under the current EU Directive, the procedures for the validation of detection methods as part of the market authorization application process for NBT plant products will in principle be the same as for the current transgenic GMOs. But a report from JRC/ENGL (2019) concluded that the validation of an event-specific detection method and its implementation for market control is not feasible for NBT plant products carrying a DNA alteration that is not unique. For instance, detection methods for those plant products that are characterized by a non-unique DNA alteration (i.e., including a targeted mutation) will probably lack the specificity required to identify the NBT plant. Since enforcement of the GMO regulations is a responsibility of member states, the EU Council requested a study from the EU Commission^1^ considering the ECJ ruling on mutagenesis breeding regarding the status of novel genomic techniques (NGTs) under Union law, and a legal proposal, if appropriate in view of the outcomes of the study. The term NGTs covers “techniques, which are capable to alter the genetic material of an organism and which have emerged or have been developed since 2001”. It also covers applications in other living organisms than plants (e.g., microorganisms and animals). In this context the EU Commission carried out a stakeholder consultation (including Euroseeds). The Commission expressed the expectation to be supplied with substantiated data. To be able to provide such substantiated data on activities of the plant breeding sector regarding the use of new breeding techniques (NBTs), Euroseeds conducted a survey within its company membership (in the following “Euroseeds survey”). The term NBTs was used in the same sense as NGTs but was limited to those applications that result in non-transgenic plants that cannot be distinguished from plants resulting from conventional breeding techniques, e.g., targeted mutagenesis and that fulfill the criteria as laid out in the Euroseeds position^2^. The survey covered the activities of companies involved in NBT-related plant research and breeding regarding three general aspects:

1. Current activities of breeding companies in view of NBT-related Research and Development (R&D) and product development
2. Future potential of NBTs for breeding companies
3. Effect of the ECJ ruling on mutagenesis breeding on breeding companies

## Materials and Methods

The Euroseeds survey was conducted between January and May 2020 by asking plant breeding companies to reply to a questionnaire. The questionnaire included mainly multiple-choice options, but also fields for free comments to provide additional information for each question. The results presented in this publication represent more than 90% replies (33 completed questionnaires) from Euroseeds direct company members involved in R&D and breeding, as well as replies (29 completed questionnaires) from company members from national seed associations across Europe. The full dataset of replies covers 62 companies. Percentages given relate either to the number of companies or to the number of total replies (in case multiple answers were possible). Companies were grouped according to annual turnover figures: the group of small companies consists of those with up to 50 Mio € turnover; medium sized companies reflect those companies with > 50 Mio up to 450 Mio € turnover and large companies were defined as those companies with > 450 Mio € turnover.

## Results

**Figure 1:**
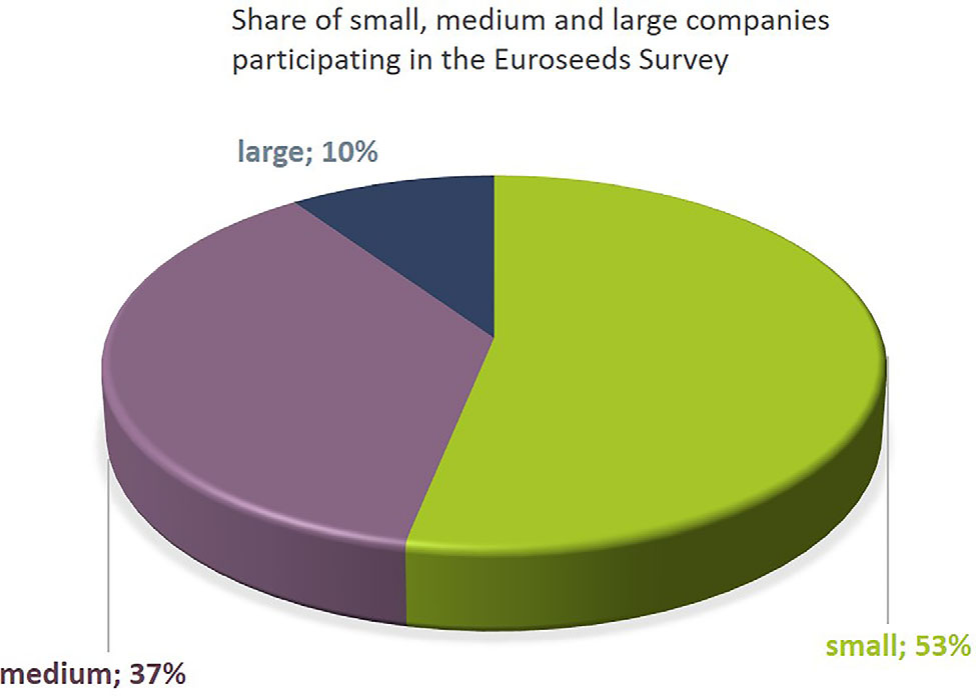
The Euroseeds Survey covers 62 plant breeding companies of all sizes. Company sizes were defined as follows: Small Companies: < 50 Mio € annual turnover; Medium-sized companies > 50 Mio € < 450 Mio € annual turnover; Large companies > 450 Mio € annual turnover.

**Figure 2:**
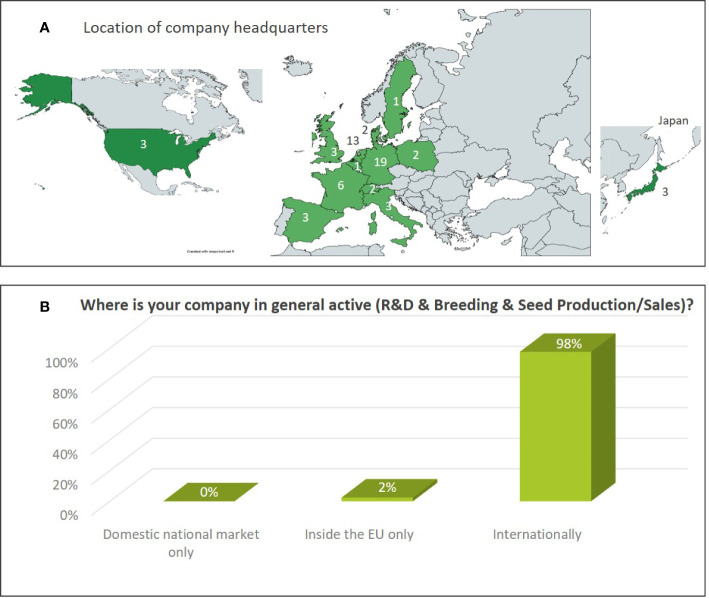
**(A)** Location of the headquarter of companies participating in the Euroseeds Survey. The figures indicate the number of company headquarters in a specific country. **(B)** Geographies in which companies active in NBT-related R&D are generally conducting their R&D, breeding or seed production/sales activities.

The dataset includes results from 10% large companies, 37% medium sized, and 53% small companies (**Figure 1**). The map shown in **Figure 2A** indicates the location of the headquarter of companies covered by the Euroseeds survey. A total of 98% of all the companies active in NBT-related R&D are acting internationally in view of either R&D, production or sales activities (**Figure 2B**). The Euroseeds survey therefore confirms the truly globalized nature of the sector and thus covers the activities of companies beyond the EU on a global level.

### Huge Interest of the Private Breeding Sector in Using NBTs

**Figure 3:**
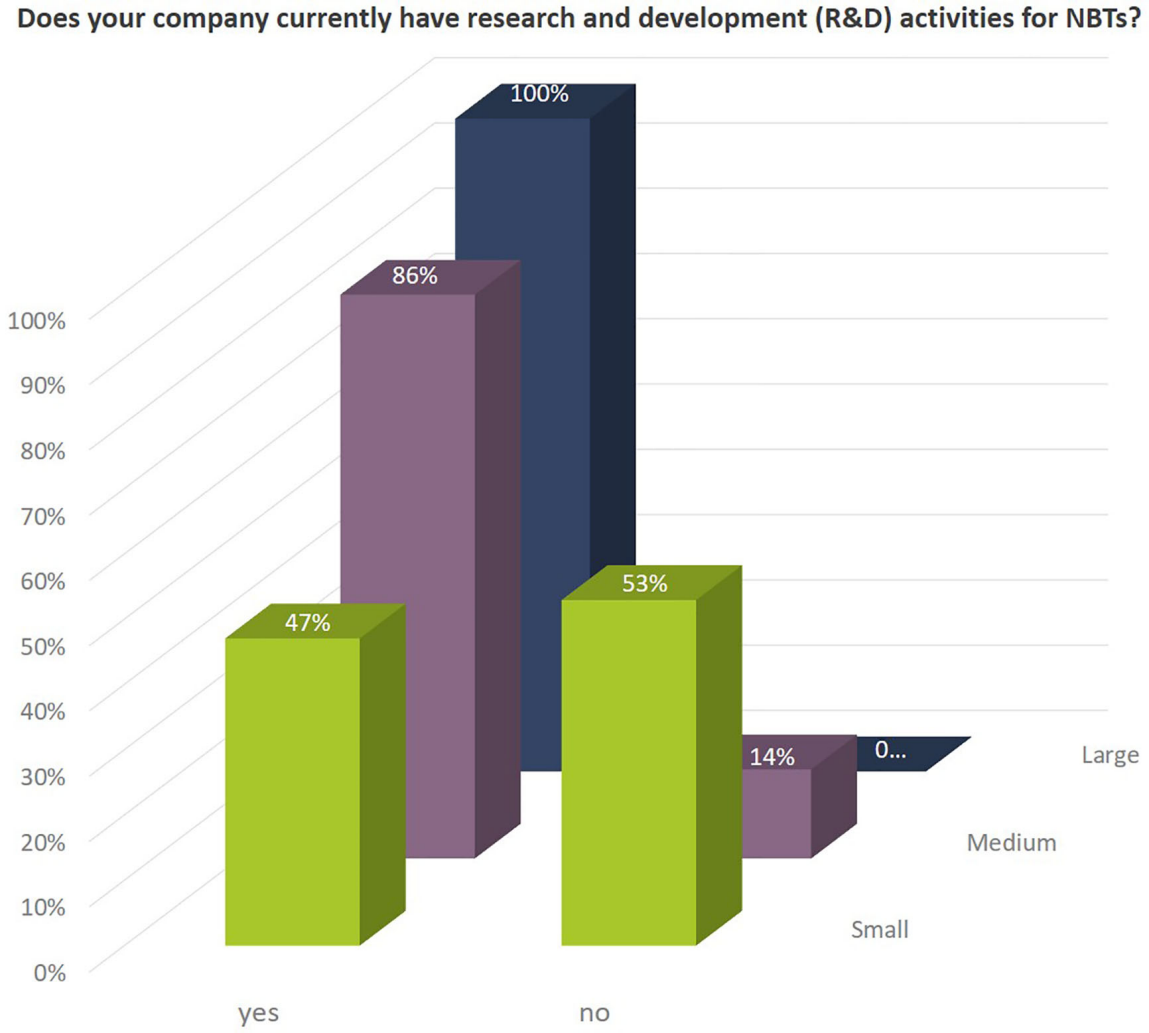
Percentage of companies according to company size currently active in NBT-related R&D.

**Figure 4:**
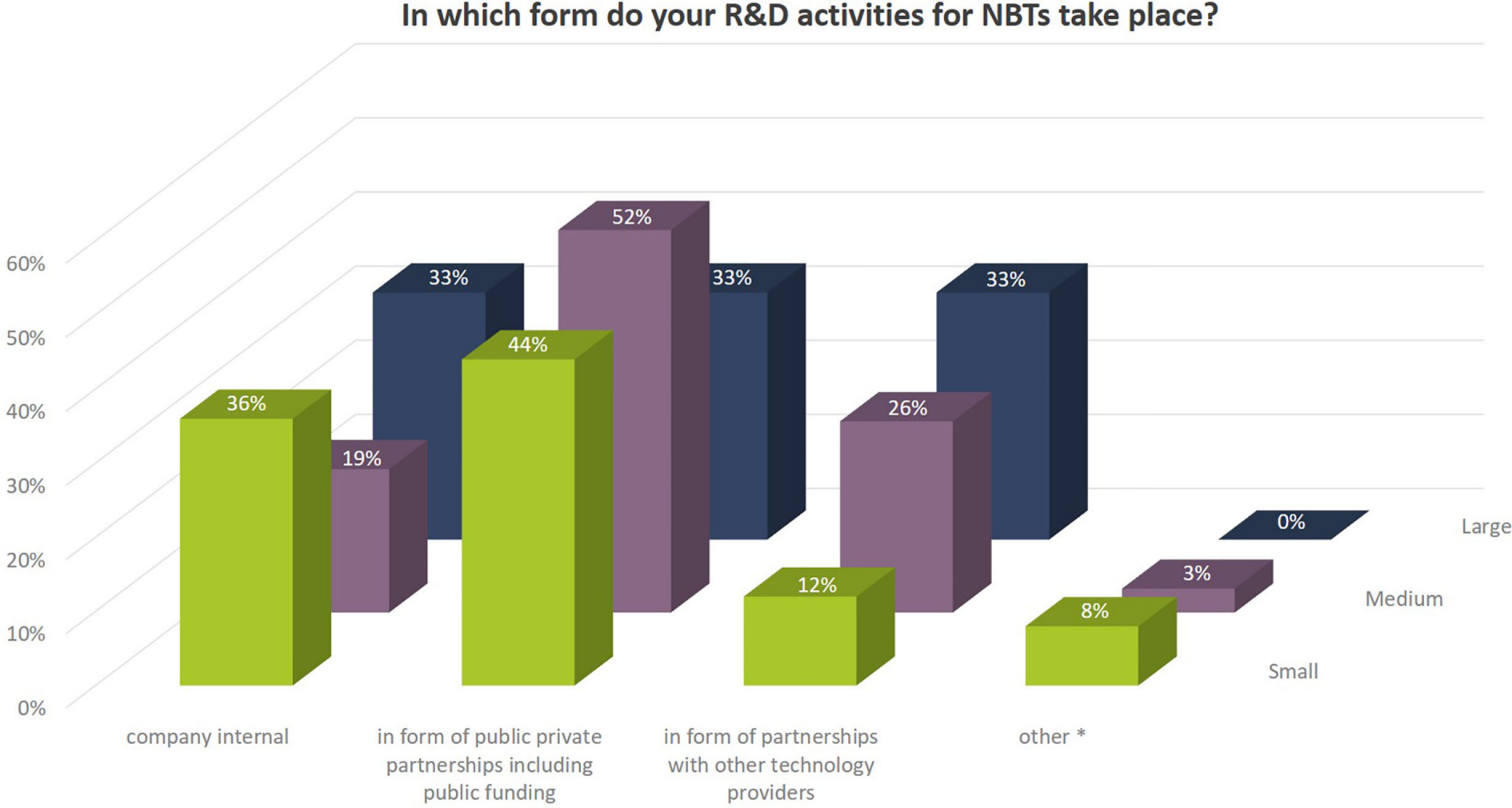
Kind of NBT-related R&D activities relate to the total number of replies of companies grouped according to company size (multiple answers possible). Total numbers of replies were: small companies, 25; medium-sized companies, 31; large companies, 18. Additional aspects mentioned by companies under “other” activities were: commissioned company-funded work at public/independent research organizations authorized for GM work, engagement through charitable funding or funding of PhD projects.

The share of small seed companies active in NBT-related R&D is close to 50% (**Figure 1**). All large companies and more than 85% of the medium sized companies are engaged in NBT-related R&D activities (**Figure 3**). The NBT-related R&D activities take place in different forms which are independent of the company size. The data suggest that SMEs rely more strongly on public private partnerships including public funding compared to larger companies (**Figure 4**).

**Figure 5:**
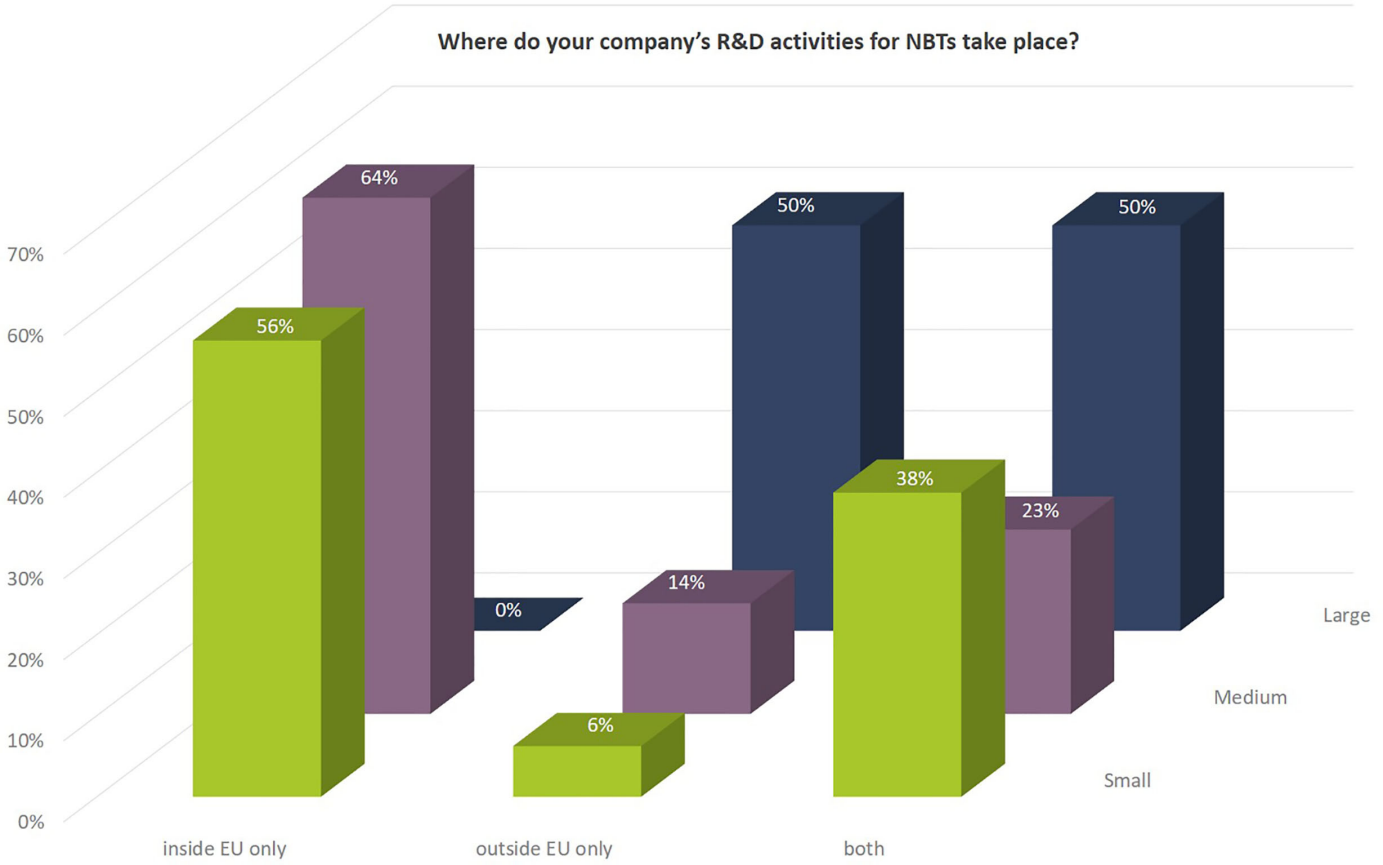
Geographies in which the company’s NBT-related R&D activities take place as to percentage of number of companies according to company size.

While the R&D activities of SMEs are more focused on Europe, larger companies are equally active in the EU as well as outside the EU (**Figure 5**).

**Figure 6:**
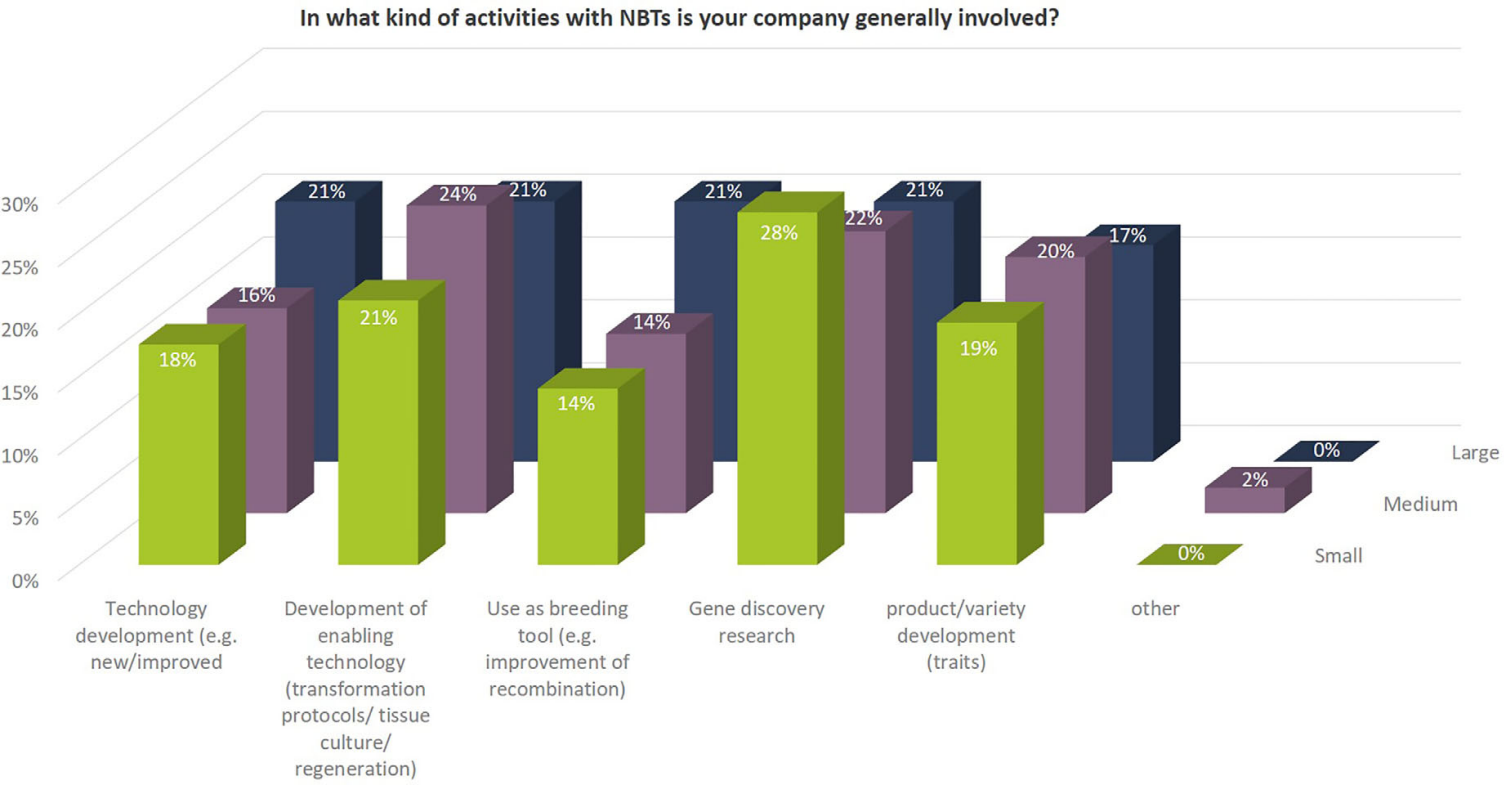
Kind of activities with NBTs in which companies are generally involved as to percentages of total replies of companies grouped according to company size (multiple answers possible). Total number of replies: small companies, 57; medium-sized companies, 49; large companies, 29. In addition, companies mentioned under “other”: Contribution to science and academic advance.

Independent of their size, many companies are involved in different kind of NBT-related R&D activities. Some of those activities concentrate on technology development (improvement of existing NBTs as such or development of new or improved enabling technologies), other activities include gene discovery research in which the NBTs are used to better understand the function of genes to be able to use the knowledge in conventional non-NBT-related breeding processes. Some SMEs mentioned that they use NBTs for implementation and improvement of existing methods only. Their main goal is to be prepared for a legislative change in the EU that would allow an economically and technically viable use of products derived from those NBTs (**Figure 6**).

**Figure 7:**
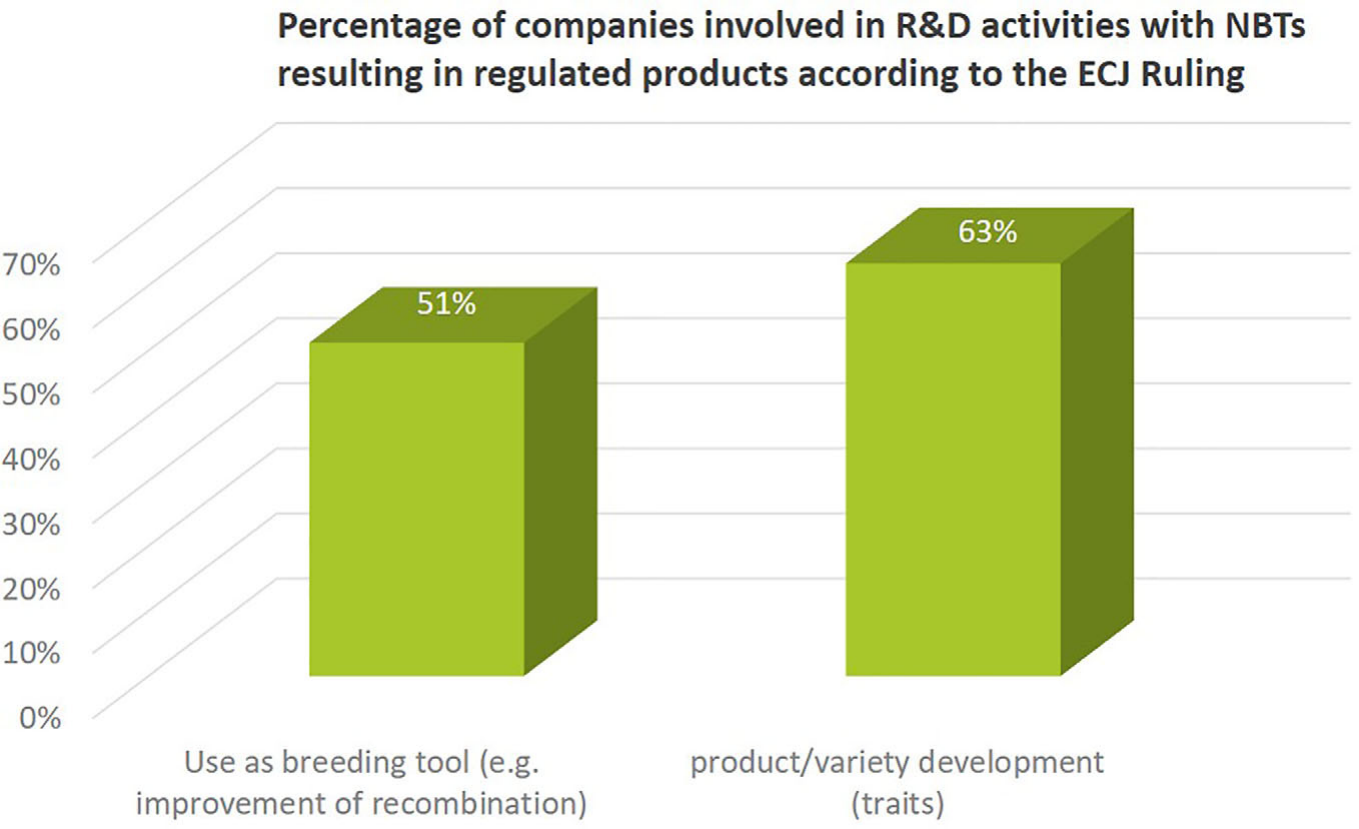
Percentage of companies (independent of size) involved in using NBTs as breeding tool or for product/variety development. Both activities would result in a product regulated as GMO in the EU after ECJ ruling on targeted mutagenesis breeding.

More than 60% of the companies active in NBT-related R&D activities also use NBTs for concrete product development or as a breeding tool (51%) (**Figure 7**). This refers to the introduction of genetic changes that lead to improved plant characteristics or to improved genetic recombination processes to increase genetic diversity in the breeding process. Since these activities (following the ECJ ruling) would lead to products regulated as GMO in the EU, some companies mentioned that their current activities are at different stages of research depending on crop type and region; some companies explicitly excluded product development activities for the EU market.

**Figure 8:**
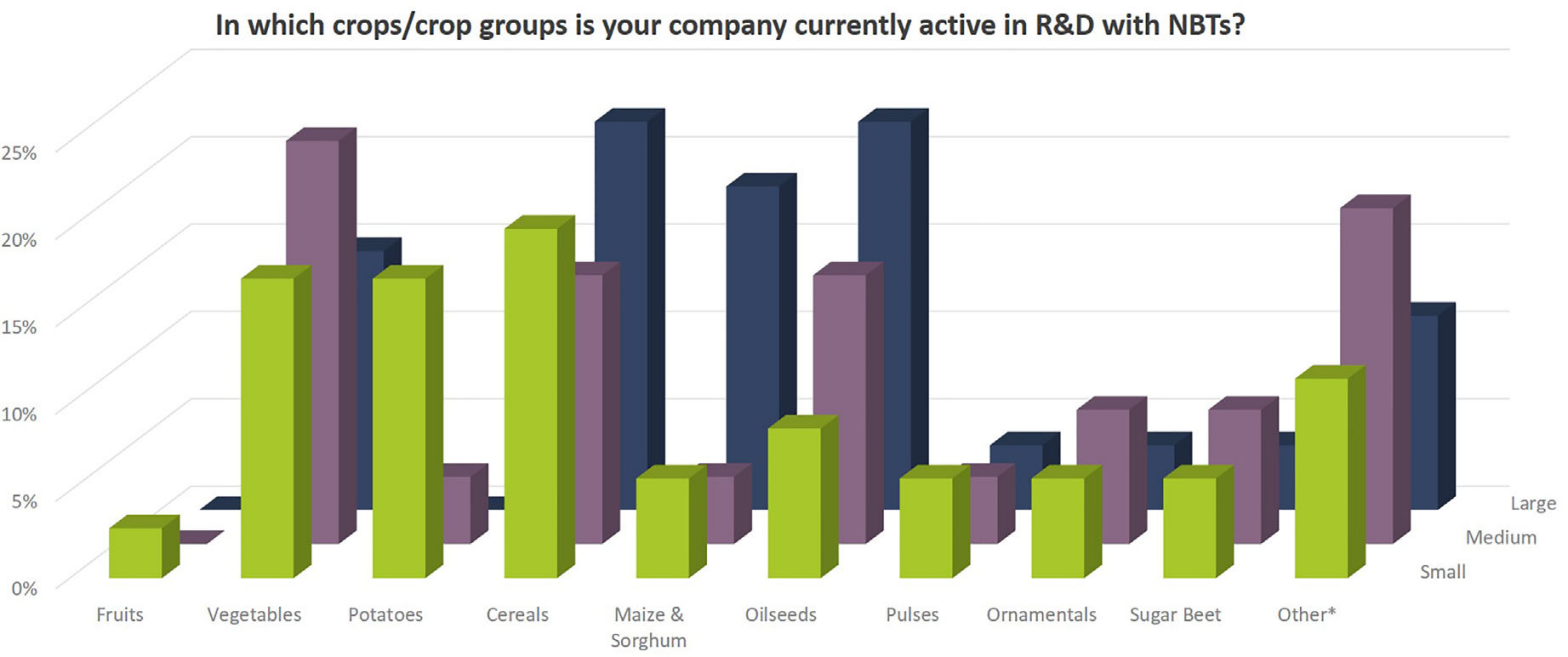
Crops/crop groups for which companies apply NBTs. Percentages relate to the total number of replies of companies grouped according to company size (multiple answers possible). Total number of replies: small companies, 35; medium-sized companies, 26; large companies, 27. In addition, companies mentioned the following crops under “other*”: soybean, cotton, rice, forage crops (grasses, legumes), chicory, model plants for gene discovery research, poppy for pharmaceutical industry, peanut, ornamentals as food and medical plants, hemp, dandelion, legumes, and stevia.

**Figure 9:**
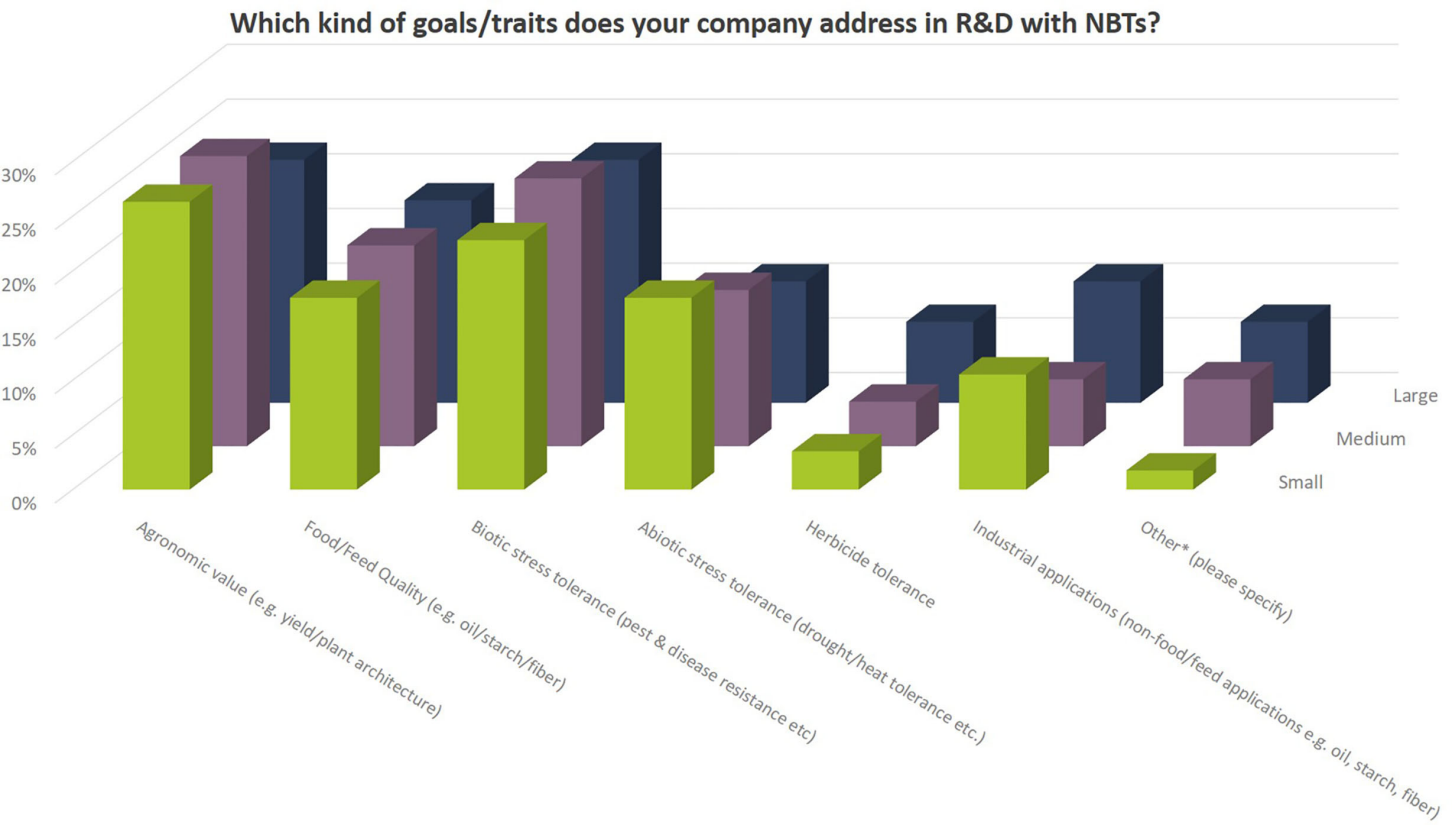
Breeding goals that companies currently address with their NBT-related R&D activities relate to the total number of replies of companies grouped according to company size (multiple answers possible). Total number of replies: small companies, 57; medium-sized companies, 49; large companies, 27. Traits mentioned under “other” relate to flavor, shelf-life, digestibility, ornamental value (flower color) and post-harvest quality.

Independent of company size, companies address a wide diversity of characteristics in the different crop species (**Figures 8**, **9**). Agronomic value (yield, plant architecture) and resistance against biotic stress (pests and diseases) are most important followed by food quality traits and abiotic stress resistance (drought, heat). Herbicide tolerance as well as industrial applications were mentioned to a minor extent only (5% and 9% respectively). Other applications include flavor related traits, shelf-life related traits, digestibility of fodder crops, ornamental value (flower color) as well as post-harvest quality (e.g., of flowers and vegetables).

**Figure 10:**
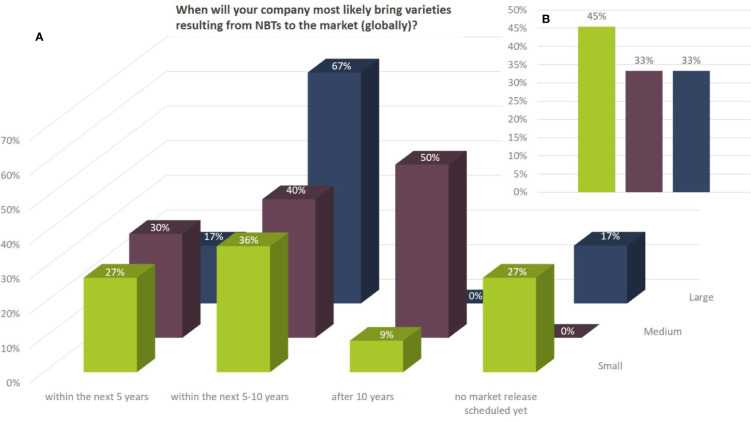
**(A)** Potential market release (globally) for products resulting from NBT-related R&D activities as to the percentage of replies according to the total number of companies per company size group (multiple answers were possible). Depending on the respective crops companies might envisage different timelines for product releases. **(B)** Percentage as to number of companies according to size that answered in addition that their intended market releases are delayed due to the current regulatory situation (e.g., GMO regulation in the EU).

Between 17% and 30% of the companies aim at bringing products to the market already within the next 5 years (globally). Most of the large companies active in NBT-related R&D are planning to bring products to the market within the next 5–10 years while 50% of the medium sized companies are intending to bring products to the market after 10 years or more. More than a quarter of the smaller companies have not projected the timeline of market releases for NBT-derived products yet (**Figure 10A**). Between 33% and 45% of the companies indicated in addition that intended market releases were postponed due to the current regulatory situation in the EU (**Figure 10B**). This indication is further substantiated in the following section.

### Biotech Regulations as Major Hurdles for Investments in NBT-Related R&D

**Figure 11:**
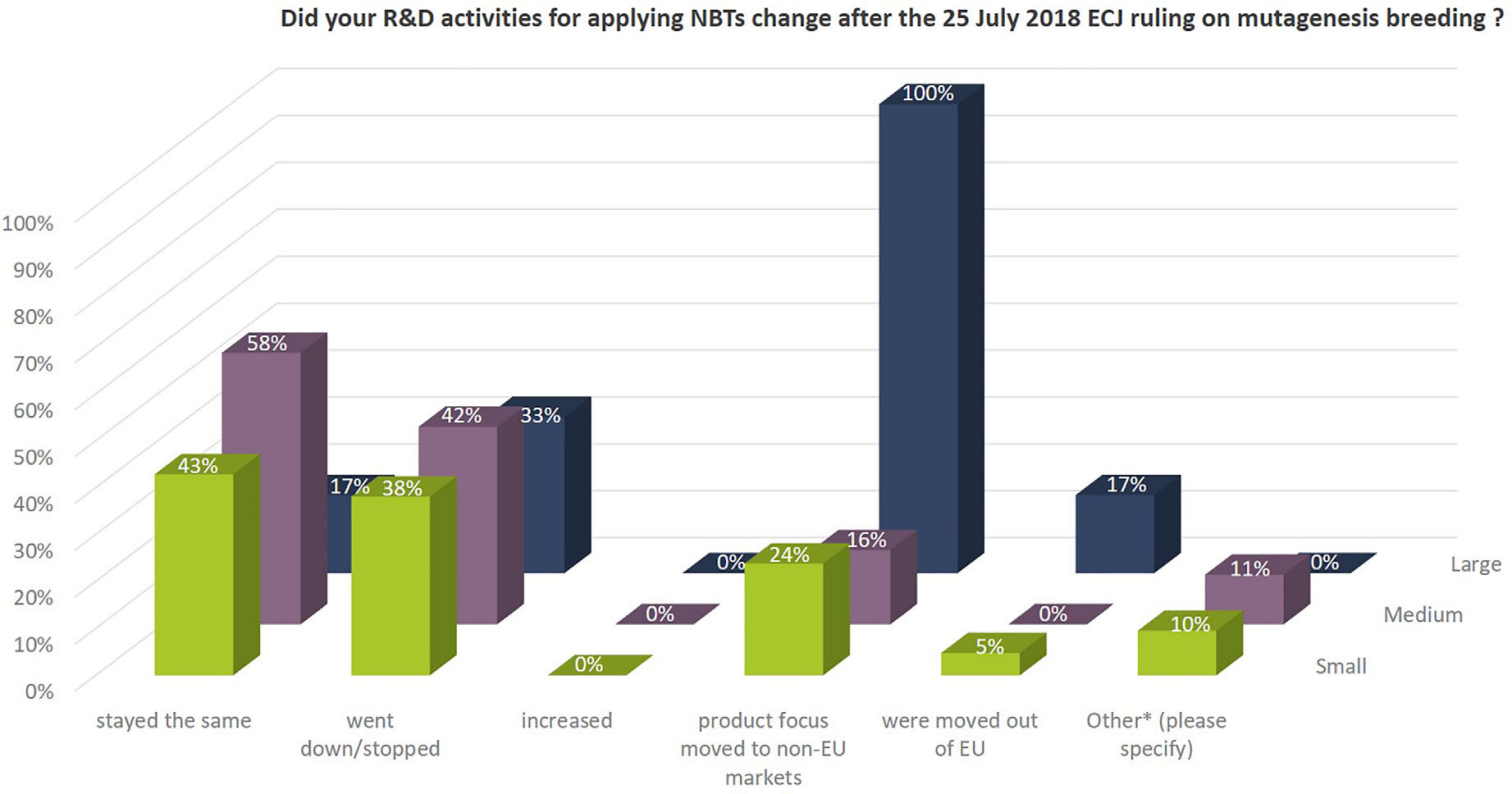
Effect of the ECJ ruling on NBT-related R&D activities of companies. Percentages as to the total number of companies per company size group. Multiple answers were possible, because the situation within companies might differ depending on the crop species and the projects. In addition, and under “other*” companies commented: all projects were reevaluated, some projects were put on hold and activities were modified in specific cases. These include discontinuation, reduction of scope, change in market focus and reevaluation of timelines; We will keep watching the future transition in the EU; some programs did not start as a consequence of the ECJ decision; After the decision of the ECJ, we decided to use the technology only for gene discovery and validation and not for product development with partners anymore.

The Euroseeds Survey asked if NBT-related R&D activities of companies changed after the 25 July 2018 ECJ ruling on mutagenesis breeding (**Figure 11**). Around 40% of the SMEs and 33% of the large companies stopped or reduced their NBT-related R&D activities after the ECJ ruling. Those companies who have major markets outside the EU moved the focus of their product development with NBTs to markets outside the EU (100% of the large and ~20% of the SMEs). In this context, it is noteworthy that NBTs are to a large extent used for gene discovery research, for technology improvement or in development of enabling technologies, which are notably not that much affected by the ruling since neither result in a regulated GMO-product according to EU Directive 2001/18. This explains why the number of SMEs that kept their NBT-related R&D activities on the same level (at least for specific projects) after the ECJ ruling is still around 50%.

**Figure 12:**
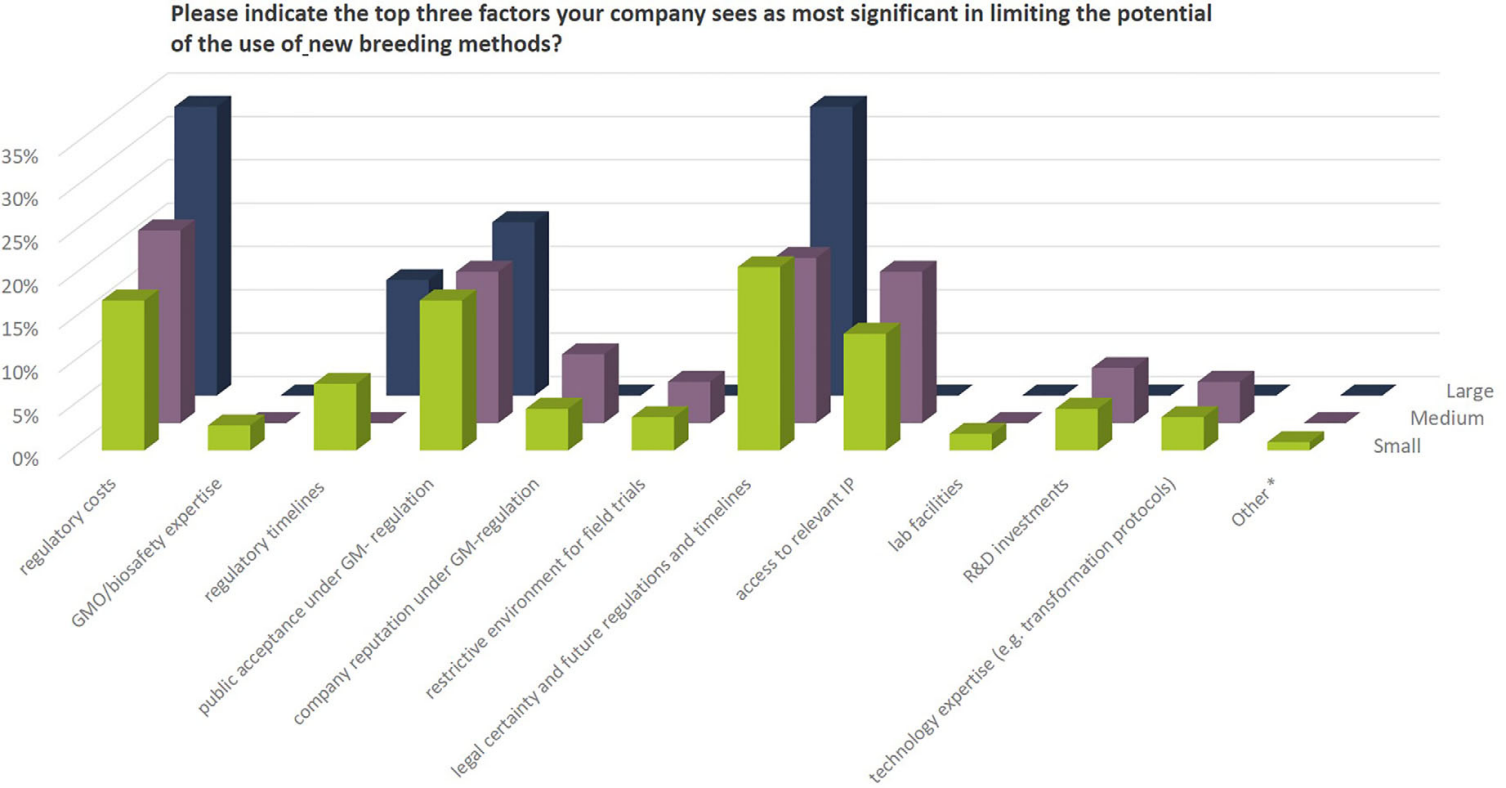
Priority factors that companies regard as most significant as to negatively influence the potential use of NBTs in their breeding programs (3 answers possible). Percentages as to the total number of replies of companies grouped according to company size (multiple answers possible). Total number of replies: small companies, 104; medium-sized companies, 63; large companies, 15. The number of replies for small companies exceeds the number of possible answers (number of small companies multiplied by three) by 2 replies, because 2 companies provided 4 replies without indicating the top three factors. The number of replies for medium and large companies is smaller than the expected. Three medium sized and one large company indicated less than three priority factors. Under “other*” one organic seed company mentioned restrictions due to private organic standards that exclude using NBTs.

From the comments provided it becomes clear that a number of companies are active in NBT-related R&D activities outside EU or for products for the non-EU market only mainly due to the regulatory situation after the ECJ ruling in 2018 (see also **Figure 11**). When it comes to decisions about investments in NBT-related R&D, prohibitive costs, long assessment and approval timelines, labeling requirements and uncertainty regarding the EU’s political decision-making process on whether NBTs would continue to be regarded as GMO’s all have a negative effect (**Figure 12**).

Companies mentioned that their R&D strategy is dependent upon market size, regulation of NBTs as well as technology readiness for each crop. For example, work on trait development is not a priority in vegetables under the current regulatory framework in the EU. Even crops like wheat and rice would in many cases not cover the high costs, considerable investment of time and uncertainty which come along with the current regulatory and political hurdles of a GM authorization process in the EU.

### Future Potential of NBTs in Plant Breeding

**Figure 13:**
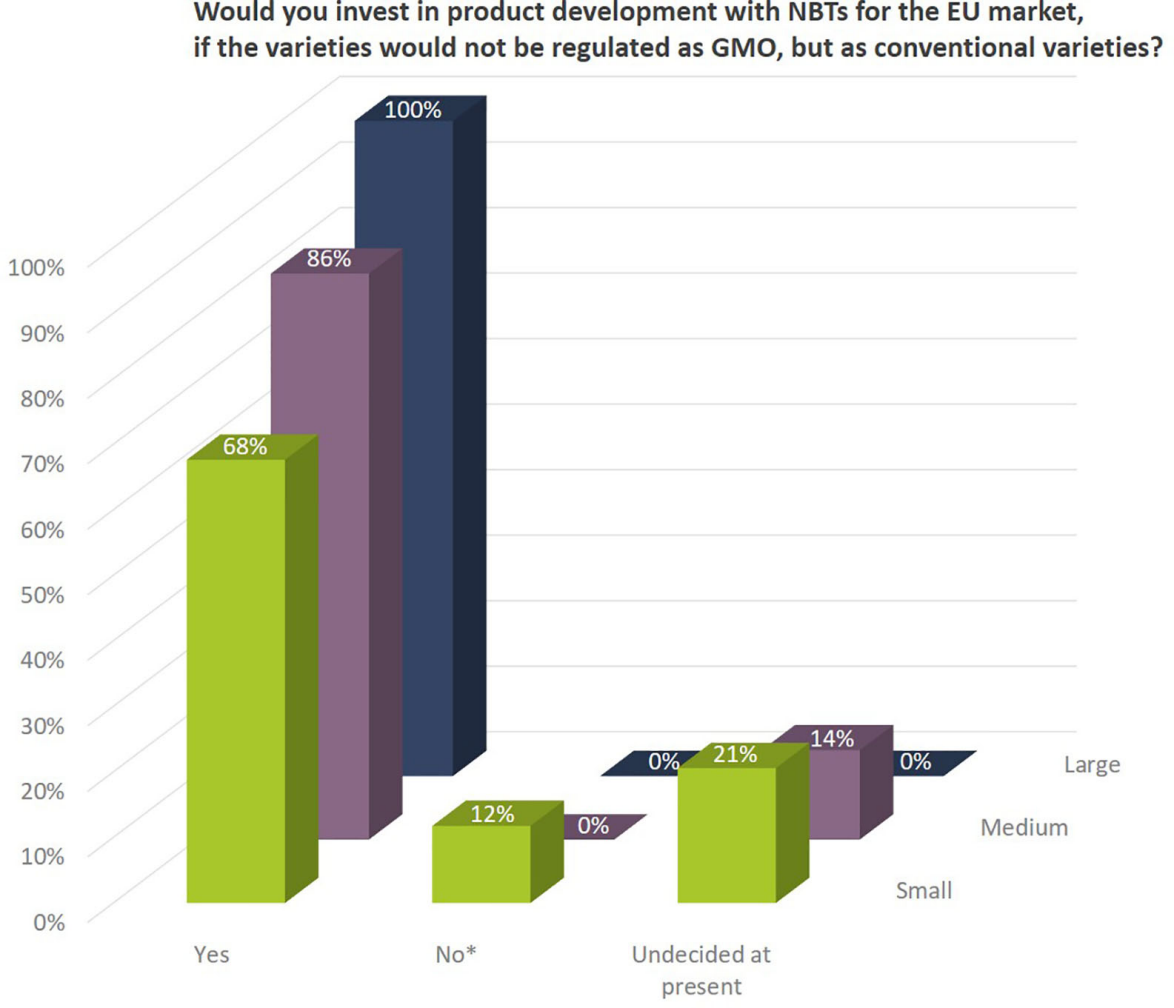
Companies’interest to invest in product development with NBTs for the EU market, if the resulting plants would not be regulated as GMOs, but as conventional varieties. Percentages as to number of companies per company size. Companies that indicated to not be interested in product development (*) were all companies with a main/exclusive focus on the organic seed market.

Independent of already ongoing activities the companies indicated that they would invest in product development with NBTs for the EU market, if the resulting plant varieties would not be regulated as GMOs, but as conventional varieties. In addition, 100% of the larger companies, 86% of the medium sized, and nearly 70% of the small companies would (further) invest in NBT-related R&D, if products would not be regulated as GMOs confirming that companies see important opportunities for these technologies (**Figure 13**).

In the context of the Euroseeds Survey companies were asked to indicate for which kind of activity and for which crops/traits they see the highest future potential for the application of NBTs. The results show that independent of their size, companies see future opportunities for NBTs in a wide range of crops and activities as well as traits. These activities include technology development (e.g., new/improved genome editing tools), development of enabling technologies (transformation protocols/tissue culture/regeneration), use of NBTs as a breeding tool, e.g., to improve recombination frequency and with that genetic diversity and for gene discovery research to better understand the function of genes. In addition, breeders see opportunities to improve agronomic value traits (yield/plant architecture), food/feed quality traits, biotic stress tolerance, abiotic stress tolerance, as well as herbicide tolerance or traits in the context of industrial applications of plants like, e.g., starch production. Other traits include improvement of nutrient use efficiency to reduce the fertilizer or water input in agriculture.

### NBT-Related Research Needs and Gaps

Especially for smaller and minor crops as well as a broad range of vegetables, the development of enabling technology (transformation protocols/tissue culture/regeneration) to apply NBTs in these crops is seen as a need for further R&D investments. This is specifically interesting for SMEs since they are more active in breeding of these smaller and niche crops.

Companies mention the need for development of enabling technology to overcome restrictions due to genotype effects or to make modern breeding techniques available for recalcitrant crops; e.g., *in vitro* regeneration is still a bottleneck for sunflower, pulses or certain cereal species.

Also, the use of NBTs as a breeding tool to generally improve genetic gain by increasing the recombination rate (Mieulet et al., 2018) and to increase genetic diversity by overcoming linkage drag is mentioned. For this, genome editing technologies might be used, but also other technologies (e.g., treatment of plants with double stranded RNA) that do not result in a permanent genetic change in the plants’ genome. These products do not show a specific characteristic resulting from the application of an NBT, but a higher general recombination rate during crossing which will result in an increased genetic variability.

The development of multiplex applications which allow addressing several alleles responsible for one characteristic or several characteristics in parallel is a clear need especially for polyploid species and for crops with long generation times like fruit trees or grape vines.

The way how NBT tools like CRISPR-Cas are delivered to the plant cell may involve an intermediate step including the use of recombinant DNA. More recent developments focus on adopting DNA-free systems to deliver the genome editing elements. DNA-free systems encounter two major problems, the severity of which may differ depending on the plant species: (i) Delivery through the plant cell wall and (ii) regeneration of plants from tissue culture cells or protoplasts (Metje-Sprink et al., 2019). Therefore, future research also needs to include the development of reliable DNA-free genome editing systems for diverse crop species. Also, a combination of genome editing applications with the double haploid (DH) technology is of huge interest. Homozygous pure DH lines could help to achieve the desired trait improvement within two generations, thus bypassing the lengthy procedure of repeated crossing and backcrossing used in conventional breeding for integrating a desirable trait into elite commercial backgrounds (Wang et al., 2019).

The improvement and additional development of new genome editing tools like base editor technologies was mentioned as a future need as well (Monsur et al., 2020).

## Discussion

**Figure 14:**
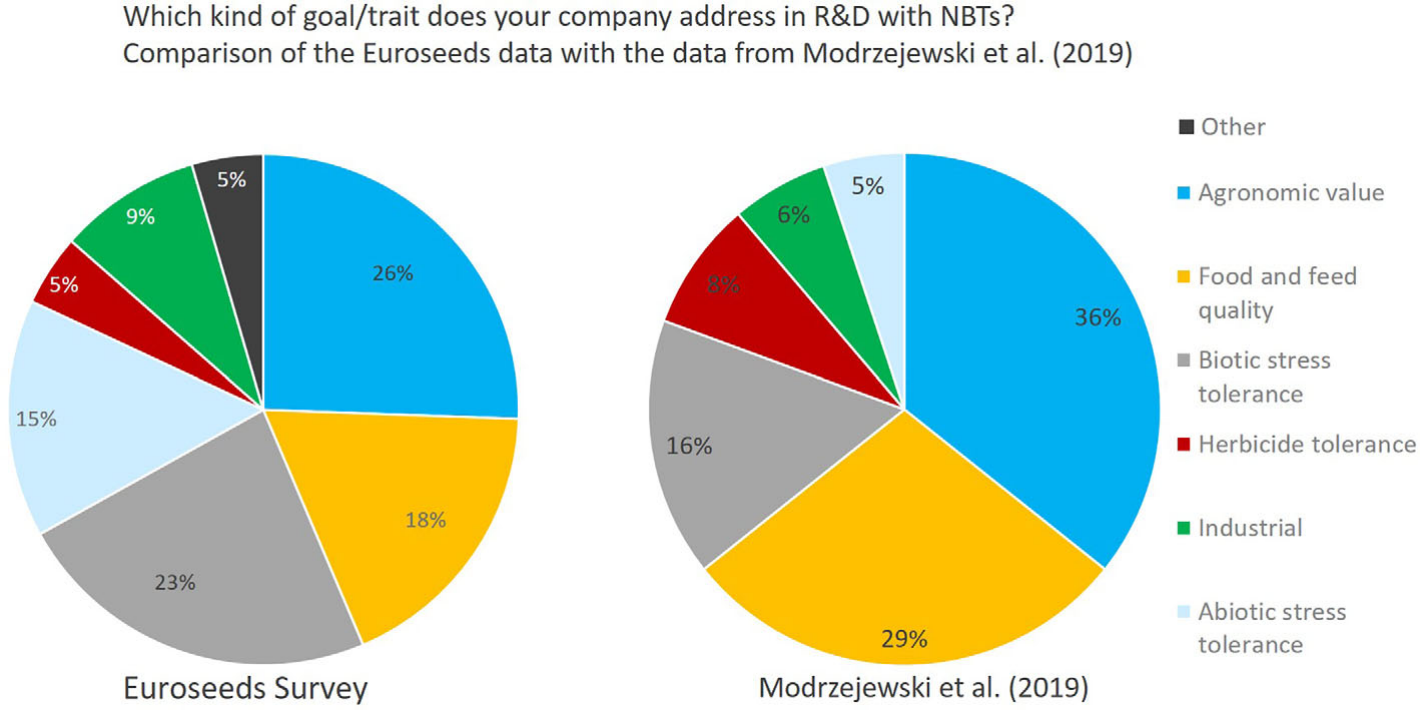
Comparison Euroseeds results as to activity of companies in NBT-related R&D with respective breeding goals compared to the results from Modrzejewski et al. (2019) which shows an assessment of market-oriented applications of crops with nutritionally, agriculturally or industrially relevant traits from published studies between January 1996 and May 2018. Traits mentioned under “other” relate to flavor, shelf-life, digestibility, ornamental value (flower color), and post-harvest quality.

The data collected by the Euroseeds survey concerning characteristics addressed by applying NBTs are comparable to the study of Modrzejewski et al. (2019). Compared to the data from Modrzejewski et al. (2019) which predominantly cover applications published in scientific journals, companies put a stronger focus on biotic and abiotic stress tolerance in their NBT-related R&D (**Figure 14**). Especially the improvement of biotic stress resistance is driven by the growing lack of availability of pesticide active substances as well as the intended further reduction of pesticide use by 50% which is one of the major strategic goals of the EU Commission as laid out in its EU Commission Communication (2020) on the Farm to Fork Strategy.

In the Euroseeds survey, companies were asked to indicate the top three factors they see as most significant in limiting the potential of the use of new breeding methods.

These are:

- Regulatory costs and timelines under the current EU GM-legislation
- uncertainty of future regulatory oversight including timelines for product approvals
- Public acceptance under GMO regulation

These issues are acknowledged also by leading scientists in the field confirming that the high level of regulatory uncertainty and differences between countries represent a bottleneck in harnessing NBTs like CRISPR technology for crop improvement (Zhang et al., 2020).

Access to relevant intellectual property (IP) was mentioned as a potential limiting factor by SMEs while this is not a priority issue raised by large companies. A number of the IP owners of genome editing technology have declared their commitment to provide broad access to the technology through non-exclusive licenses. While this would still require individual agreements between business partners, the academic, non-commercial use of the technologies appear to be readily accessible (Rozen, 2017).

Some SMEs also mention limited resources when it comes to technology expertise or R&D investments in their companies. For these companies the opportunity to use NBTs in public private partnerships is of importance.

The negative impact of the ECJ ruling on R&D activities for commercial product development is actually higher than suggested by the pure number of companies as it is mainly bigger companies with respective big R&D spending that have moved activities outside the EU. Having R&D facilities outside the EU allows more flexibility when it comes to transferring NBT-related R&D activities including field trials to geographies with enabling regulatory environments. This is confirmed by a study from University of Wageningen scientists who did a survey among Dutch breeding companies showing that companies with major markets outside the EU intend to reallocate their research (Wesseler et al., 2019). The European Green Deal and its Farm to Fork Strategy requires EU agriculture to become more sustainable by reducing the amount of pesticides by 50%, and the amount of fertilizers by 20% until 2030 while taking 10% of farmland out of production and increasing the EU organic farming area with the aim to achieve 25% of total farmland under organic farming by 2030 (EU Commission Communication, 2020). Although plant breeding has successfully contributed to a more sustainable crop production and biodiversity in the past (Noleppa, 2016), all these measures will create a productivity gap in food production and the combination of objectives can only be achieved by means of sustainable intensification, which includes the genetic improvement of plants. Otherwise the EU might become more dependent on agricultural imports. Conventional plant breeding may still be able to address some of these challenges effectively, but it is more time-consuming. NBTs have the potential to address these drawbacks (Aerni, 2019).

For the majority of SMEs which have their major market within the EU, moving their research or their product focus to non-EU markets is not an option. Many NBT-related research projects within companies were re-evaluated, some were put on hold and activities were modified in specific cases. This includes discontinuation of projects, reduction of scope, change of market focus and re-evaluation of timelines. Also, some projects did not start as a consequence of the ECJ ruling in 2018. The Dutch study confirms a strong negative effect of the ECJ ruling on the investments in CRISPR-Cas technology especially for the vegetable sector (Wesseler et al., 2019).

The regulatory uncertainty pertaining to products of NBTs is not due to scientific concerns, but rather political interference in the regulatory approval process. Given the highly competitive market for strategic agricultural and food investments, the level of uncertainty that exists within the EU has the potential to divert potential research and development investments away from the EU to markets with more science-based, risk-proportionate, and innovation-supporting regulations (Lassoued et al., 2018). Uncertainty and irreversibility have a strong effect on postponing investment in R&D with NBTs (Purnhagen and Wesseler, 2019).

The data of the Euroseeds survey confirm this. Around 40% of the companies who want to bring products to the market delayed their intended market release due to regulatory reasons (e.g, the current GMO regulation in the EU, the lack of international harmonization, the lack of legal certainty until 2018 (ECJ ruling) as well as the ongoing uncertainty of future regulatory oversight). This shows that the regulatory situation in Europe already has an impact on NBT-related R&D as well as the innovation capacity and product development also on a global level.

In addition, companies mentioned that their R&D strategy is dependent upon market size, regulation of NBTs as well as technology readiness for a particular crop. For instance, the work on trait development is not a priority for vegetables under the current regulatory framework in the EU. In many cases even large commodity crops like wheat and rice may not recoup the high bring-to-market costs which go along with the regulatory hurdles for GM crops.

Since larger companies have a higher share of R&D facilities outside the EU, it is easier for them to also benefit from the use of NBTs for concrete product development outside of the EU market thereby increasing their readiness to restart development for the EU market should the regulatory environment change at a future point in time. In this respect, the current restrictive regulatory regime in the EU will provide larger companies with a head start in case the EU legislation would become more permissive. The consequence is that especially SMEs lag behind while large companies can continue developing and applying NBTs in other parts of the world with more enabling regulations. Specifically, SMEs have less infrastructure available and thus less flexibility in moving R&D programs between regions. Whelan et al., 2020 analyzed the situation for Argentina which does exempt certain non-transgenic NBT product from their biotech regulations and concluded that NBT products development is driven by a more diverse group of developers and led mostly by small and medium-sized enterprises (SMEs) and public research institutions.

Countries currently have different systems to evaluate and regulate products entering the market, as for example, Genetically Modified Organisms (GMOs). This creates a patchwork of national regulations: some countries regulate specific technologies (like the EU), while others regulate based on the characteristics of the final product or both (Schmidt et al., 2020). Furthermore, although most countries which already implemented or discussed new or updated policies, base their evaluation on the absence or presence of a novel combination of genetic material as laid out in the Living Modified Organism (LMO) definition of the Cartagena protocol (Cartagena Protocol on Biosafety 2000), definitions for “GMO”, “biotechnology”,”genetic engineering”, and “bioengineering” are still inconsistent across countries (Jorasch, 2019). In view of the international situation with different regulatory policies in place, the challenge for SMEs to comply with these diverse requirements is higher than for large companies. This again reduces the competitiveness of EU SMEs (Jorasch, 2020).

Most breeders worldwide make use of the so-called “breeders’ exemption” as provided for in UPOV-based plant variety protection laws. This allows breeders free access to competitor’s commercial germplasm for further breeding and with this to build their breeding efforts on the innovations of other breeders. This breeders’ exemption highly contributes to the innovative strength of the breeding sector (UPOV, 2009). EU breeders will be forced to restrict themselves from access to genetic diversity from certain jurisdictions for conventional cross breeding programs in order to avoid unintentional integration of genetic material from organisms which are qualified as GMOs in the EU (but not necessarily elsewhere). This will lead to two restriction effects: firstly, less access to general genetic diversity by not being able to use commercial germplasm from competitors (breeders’ exemption) or from research collaborations for conventional cross-breeding and secondly, no access to new genetic diversity and interesting traits developed *via* NBTs in other parts of the world with a more enabling regulatory environment.

The current regulatory situation in the EU, especially the lack of GM field trial capacities, was mentioned as a bottleneck for the application and optimization of NBTs. This also negatively affects gene discovery research activities since the effect of the function of genes on the plant phenotype often needs to be checked under field conditions.

Also, there is uncertainty among young researchers about the impact of the court ruling for future perspectives in applied plant sciences in Europe. This is illustrated by the fact that different groups of young researchers have started campaigns with the goal to enable the use of genome editing for sustainable agriculture and food^3^. If NBT-related public research in Europe is negatively affected by the current regulatory situation this also has a negative impact on the seed sector since these young scientists often are the future employees of these companies. Public support of basic research for NBTs is equally important especially in view of the further development of the NBTs and their applicability to a wide range of species. In this context the financial support of genome research including whole genome sequencing of recalcitrant crops is important.

The negative effect of disproportionate regulatory requirements on public investment in breeding is also confirmed in Canada by a study which concluded that public breeders have had limited capacity to apply transgenic breeding techniques within their programs due to the additional time and cost required to receive regulatory approval (Gleim et al., 2020).

In view of potential societal/consumer concerns most studies conclude that attitude and acceptance change with knowledge, which shows the need for balanced information and the importance of science as well as risk communication (Marie et al., 2020). It is the responsibility of all stakeholders including authorities, to translate science into laymen language and with that facilitate informed decisions of consumers and informed political debates.

The outcome of the Euroseeds survey regarding the future potential of NBTs in plant breeding is confirmed by a study with Canadian plant breeders. They highlighted different aspects of precision breeding with CRISPR-Cas9. These include precision editing without disruption to the remainder of the genome, the confirmation of genes of interest (which the Euroseeds survey addresses as gene discovery research), cost reduction, and the recent democratization (improved freedom to operate) of CRISPR-Cas9. The study also highlights that precision-breeding capabilities stand out as benefits, allowing plant breeders an increasingly greater ability to target and control the intended mutations. By far the most significant benefit recognized by 90% of all respondents was the potentially reduced regulatory oversight of CRISPR-derived varieties, mostly in comparison to transgenic GM breeding technologies in Canada (Gleim et al., 2020).

Our data indicate that the potential benefit of applying NBTs in breeding extends far beyond a few cash crops and revenue-generating traits but will potentially also deliver improved niche and minor crops harboring characteristics that support the goals of the European Green Deal and its Farm to Fork Strategy. Companies of all sizes are preparing and stand ready to enter into this endeavor also in Europe. However, the regulatory system in the EU needs to develop towards a more enabling environment for the potential benefits of NBTs to materialize in Europe.

## Author Contributions

The author confirms being the sole contributor of this work and has approved it for publication. Raw data for the study were provided by the companies as mentioned in the publication.

## Conflict of Interest

The author is employed by Euroseeds which is an industry umbrella organization to which the companies that provided the data to the study are members. All individual company data were treated confidentially by Euroseeds. The author was at no point in time during the analysis of the data and the preparation of the manuscript influenced by the data providing companies or any other third party.

## Footnotes

1 Council Decision (EU) 2019/1904 of 8 November 2019 https://eur-lex.europa.eu/legal-content/EN/TXT/?uri=CELEX:32019D1904 (accessed 29-06-2020).

2 https://www.euroseeds.eu/app/uploads/2019/07/18.1010-Euroseeds-PBI-Position-1.pdf (accessed 29-06-2020) 1) there is no novel combination of genetic material, i.e., there is no stable insertion in the plant genome of one or more genes that are part of a designed genetic construct or 2) the final plant product contains solely the stable insertion of inherited genetic material from sexually compatible plant species or 3) the genetic variation is the result of spontaneous or induced mutagenesis.

3 GeneSprout Initiative https://www.genesproutinitiative.com/ (accessed 17-06-2020); Give CRISPR a Chance https://www.sciencefordemocracy.org/give-crispr-a-chance-update/ (accessed 17-06-2020); Grow Scientific Progress https://www.growscientificprogress.org/ (accessed 17-06-2020).

## References

[B1] Aerni P. (2019). Politicizing the precautionary principle: why disregarding facts should not pass for farsightedness. Front. Plant Sci. 10, 1053. 10.3389/fpls.2019.01053 31507627 PMC6718141

[B2] Directive 2001/18/EC of the European Parliament and of the Council of 12 March 2001 on the deliberate release into the environment of genetically modified organisms and repealing Council Directive 90/220/EEC. Available at: https://eur-lex.europa.eu/legal-content/EN/TXT/?uri=CELEX%3A32001L0018 (Accessed 17-06-2020).

[B3] EU Commission Communication (2020). A Farm to Fork Strategy for a fair, healthy and environmentally-friendly food system, 20 May 2020. Available at: https://eur-lex.europa.eu/legal-content/EN/TXT/?qid=1590404602495&uri=CELEX%3A52020DC0381 (Accessed 16.06. 2020).

[B4] European Court of Justice (2018). Judgement on case C-528/16. Available at: http://curia.europa.eu/juris/document/document.jsf?text=&docid=204387&pageIndex=0&doclang=EN&mode=lst&dir=&occ=first&part=1&cid=5399146 (Accessed 16.06.2020).

[B5] Gleim S. Lubieniechi S. Smyth S. J. (2020). CRISPR-Cas9 Application in Canadian public and private plant breeding. CRISPR J. 3 (1), 44–51. 10.1089/crispr.2019.0061 32091256

[B6] Jorasch P. (2019). The global need for plant breeding innovation. Transgenic Res. 28, 81–86. 10.1007/s11248-019-00138-1 31321688

[B7] Jorasch P. (2020). Will the EU stay out of step with science and the rest of the world on plant breeding innovation? Plant Cell Rep. 39 (1), 163–167. 10.1007/s00299-019-02482-2 31754780

[B8] JRC/ENGL (2019) Detection of food and feed plant products obtained by new mutagenesis techniques. Available at: https://gmo-crl.jrc.ec.europa.eu/doc/JRC116289-GE-report-ENGL.pdf (Accessed 17-06-2020).

[B9] Lassoued R. Smyth S. J. Phillips P. W. B. Hesseln H. (2018). Regulatory uncertainty around new breeding techniques. Front. Plant Sci. 9, 1291. 10.3389/fpls.2018.01291 30233627 PMC6131982

[B10] Lusser M. Parisi C. Rodriguez Cerezo E. Plan D. (2011). New plant breeding techniques. State-of-the-art and prospects for commercial development. JRC Sci. Tech. Rep. JRC63971. 10.2791/54761

[B11] Marie A. Altay S. Strickland B. (2020). The cognitive foundations of misinformation on science. EMBO Rep. 21, e50205. 10.15252/embr.202050205 32249542 PMC7132178

[B12] Metje-Sprink J. Menz J. Modrzejewski D. Sprink T. (2019). DNA-free genome editing: past, present and future. Front. Plant Sci. 9, 1957. 10.3389/fpls.2018.01957 30693009 PMC6339908

[B13] Mieulet D. Aubert G. Bres C. Klein A. Droc G. Vieille E. (2018). Unleashing meiotic crossovers in crops. Nat. Plants 4, 1010–1016. 10.1038/s41477-018-0311-x 30478361

[B14] Modrzejewski D. Hartung F. Sprink T. Krause D. Kohl C. Wilhelm R. (2019). What is the available evidence for the range of applications of genome-editing as a new tool for plant trait modification and the potential occurrence of associated off-target effects: a systematic map. Environ. Evid. 8, 27. 10.1186/s13750-019-0171-5

[B15] Monsur M. B. Shao G. Lv Y. Ahmad S. Wei X. Hu P. (2020). Base editing: the ever expanding clustered regularly interspaced short palindromic repeats (CRISPR) Tool Kit for Precise Genome Editing in Plants. Genes (Basel) 11 (4), 466. 10.3390/genes11040466 PMC7231171 32344599

[B16] Noleppa S. (2016). The economic, social and environmental value of plant breeding in the European Union. An ex post evaluation and ex ante assessment. HFFA Research Paper 03/2016. Available at: http://www.plantetp.org/system/files/publications/files/hffa_research_paper_plant_breeding_eu.pdf (Accessed 02-07-2020).

[B17] Purnhagen K. P. Wesseler J. H. H. (2019). Maximum vs minimum harmonization: what to expect from the institutional and legal battles in the EU on gene editing technologies, published online in Wiley Online Library. Available at: https://onlinelibrary.wiley.com/doi/epdf/10.1002/ps.5367 (Accessed 17-06-2020). 10.1002/ps.5367 PMC6767570 30714289

[B18] Rozen I. (2017). Removing a major CRISPR licensing roadblock in agriculture. Available at: https://www.broadinstitute.org/news/removing-major-crispr-licensing-roadblock-agriculture (Accessed 30-06-2020).

[B19] Schmidt S. Belisle M. Frommer W. B. (2020). The evolving landscape around genome editing in agriculture. EMBO Rep. 21, e50680. 10.15252/embr.202050680 32431018 PMC7271327

[B20] UPOV (2009). Responding to the challenges of a changing world: The role of new plant varieties and high quality seed in agriculture; Proceedings of the second World Seed Conference, UPOV Publication No. 354(E).

[B21] Wang B. Zhu L. Zhao B. Zhao Y. Xie Y. Zheng Z. (2019). Development of a haploid-inducer mediated genome editing system for accelerating maize breeding. Mol. Plant 12, 597–602. 10.1016/j.molp.2019.03.006 30902686

[B22] Wesseler J. Politiek H. Zilberman D. (2019). The economics of regulating new plant breeding technologies - implications for the bioeconomy illustrated by a survey among dutch plant breeders. Front. Plant Sci. 10, 1597. 10.3389/fpls.2019.01597 31921246 PMC6932994

[B23] Whelan A. I. Gutti P. Lema M. A. (2020). Gene editing regulation and innovation economics. Front. Bioeng. Biotechnol. 8, 303. 10.3389/fbioe.2020.00303 32363186 PMC7181966

[B24] Zhang Y. Pribil M. Palmgren M. Gao C. (2020). A CRISPR way for accelerating improvement of food crops. Nat. Food 1, 200–205. 10.1038/s43016-020-0051-8

